# Effects of the Roasting-Assisted Aqueous Ethanol Extraction of Peanut Oil on the Structure and Functional Properties of Dreg Proteins

**DOI:** 10.3390/foods13050758

**Published:** 2024-02-29

**Authors:** Sicheng Wang, Yubao Guo, Xiuling Zhu, Dan Xie, Zhenzhen Wang

**Affiliations:** School of Biological and Food Engineering, Anhui Polytechnic University, Wuhu 241000, China

**Keywords:** peanut, roasting, aqueous ethanol, functional property, structural characterization

## Abstract

The effects of the roasting-assisted aqueous ethanol extraction of peanut oil on the structure and functional properties of dreg proteins were investigated to interpret the high free oil yield and provide a basis for the full utilization of peanut protein resources. The roasting-assisted aqueous ethanol extraction of peanut oil obtained a free oil yield of 97.74% and a protein retention rate of 75.80% in the dreg. The water-holding capacity of dreg proteins increased significantly, and the oil-holding capacity and surface hydrophobicity decreased significantly, reducing the binding ability with oil and thus facilitating the release of oil. Although the relative crystallinity and denaturation enthalpy of the dreg proteins decreased slightly, the denaturation temperatures remained unchanged. Infrared and Raman spectra identified decreases in the C-H stretching vibration, Fermi resonance and α-helix, and increases in random coil, β-sheet and β-turn, showing a slight decrease in the overall ordering of proteins. After the roasting treatment, 62.57–135.33% of the protein functional properties were still preserved. Therefore, the roasting-assisted aqueous ethanol extraction of peanut oil is beneficial for fully utilizing the oil and protein resources in peanuts.

## 1. Introduction

Peanuts are one of the most important oilseeds in the world, with high oil (44–56%) and protein contents (22–30%) [1]. Peanut protein has high nutritional value, comparable to animal proteins such as in meat and eggs [2]. Peanut protein has a comprehensive amino acid composition and is a good source of arginine. Based on solubility, peanut protein can be divided into water-soluble and salt-soluble proteins, and salt-soluble proteins include arachin and conarachin [3].

Studies have shown that peanut protein has good functional properties, such as solubility, a water(oil)-holding capacity, emulsification activity, emulsification stability, foaming ability, foam stability and surface hydrophobicity [4], and it is widely used in the food industry. Peanut protein with a high water(oil)-holding capacity can be used to make meats, sausages, bread and cakes [5]. The high emulsifying activity of peanut protein can be used to manufacture ice cream, beverages, soup stocks and salad dressings. Protein isolate, protein concentrates and the dreg of peanut all have functional properties similar to those of peanut protein and can be used to prepare emulsifiers and foaming agents [6]. In addition, arachin can be used to prepare foodborne antihypertensive peptides [7]. Therefore, peanut protein has a high utilization value. In this way, it is possible to ensure the source of protein raw materials by avoiding the waste of peanut protein resources due to denaturation.

The traditional methods of mechanical pressing and solvent extraction for preparing peanut oil have disadvantages such as severe protein denaturation, a low oil yield, and harmful solvent residue in product oil, resulting in a waste of protein resources [8]. In contrast, using the aqueous substitution method to simultaneously separate peanut oil and protein results in a lower degree of protein denaturation, but it is accompanied by severe emulsification, a lower free oil yield, and a higher residual oil rate of up to 10% [9]. In using the aqueous enzyme method to improve the oil extraction yield and reduce the demulsification, the free oil yield can reach 95%, and the yield of peanut protein can reach about 75% [10,11]. However, the cost of enzymes is high, and bitter peptides are probably produced. It is necessary to develop an aqueous extraction process of peanut oil that does not use biological enzymes and does not require additional demulsification.

Research has shown that moderate roasting can improve the yields of free oil and protein from rapeseed to a certain extent [12], and the functional properties of rapeseed protein can also be well maintained [5]. When peanut oil was extracted from roasting pretreated oilseeds using the aqueous substitution method, the free oil yield increased from 50.95% to 63.50% [13]. When oil and protein hydrolysates were extracted simultaneously by using a 2% enzyme solution from roasted peanuts, the free oil yield increased from 68.80% to 78.60% [14]. In addition, ethanol as an environmentally friendly extractant has a positive effect on reducing demulsification [15]. Grape seed oil has been extracted using a 45% aqueous ethanol solution, resulting in a free oil yield of 86.57% [16]. Therefore, the roasting-assisted aqueous ethanol extraction of peanut oil is expected to efficiently obtain free oil, maintain the functional properties of proteins and realize the rational utilization of peanut resources. This study investigated the changes in structure and functional properties of dreg proteins after an efficient extraction of free oil from peanuts using roasting-assisted aqueous ethanol, to provide a basis for the rational utilization of peanut resources. From the perspective of the structure, functional properties and microscopic morphological changes in peanut dreg proteins, this study tried to explain the mechanism of roasting-assisted aqueous ethanol extraction to improve the free oil yield. The roasting-assisted aqueous ethanol extraction process is efficient, green and environmentally friendly, which is of great significance for the full utilization of peanut resources.

## 2. Materials and Methods

### 2.1. Materials and Reagents

Peanuts were purchased from the City Celebrity Supermarket (Wuhu, Anhui, China). The Soxhlet extraction method (AOAC Method, 995.19) [17], air oven method (AOAC Method, Ca 2c-25) [18] and Kjeldahl method (AOAC Method Ac 4-91) [19] were used to determine the oil contents, moisture contents and protein contents, respectively. The peanuts used in the present study were initially composed of 49.40% ± 0.67% oil, 3.80% ± 0.20% moisture and 22.71% ± 0.39% protein. Ethanol and potassium phosphate dibasic of analytical grade, and potassium bromide and barium sulfate of spectral purity were purchased from Sino Pharm Chemical Reagent Co., Ltd. (Shanghai, China). Oil red O was purchased from Wokai Bioengineering Co., Ltd. (Shanghai, China).

### 2.2. Peanut Roasting and Grinding

Peanut kernels (50 g) were weighed, roasted at 180 °C for 10 min (pre-experimental determination), cooled to a room temperature of 25 °C and kneaded to remove the testa. Peeled peanut kernels without roasting were taken as the control. Peanut kernels (peeled, 50 g) were weighed and then ground for 20 s once at 24,000 r/min using a high-speed universal grinder (FW-100, Tester Corporation, Tianjin, China), repeated five times. The obtained peanut paste was sealed and refrigerated for later use.

### 2.3. Oil Extraction and Dreg Preparation

Peanut paste (5 g) was evenly mixed with a 45% aqueous ethanol solution in a liquid-to-material ratio of 3:1 (mL/g) in a conical flask. Subsequently, the sample was shaken using a thermostatic water-bath vibrator (SHA-B, Jintan Jieruier Electric Appliance Co., Ltd., Changzhou, China) at 60 °C and 140 r/min for 1.5 h. The extraction mixture was centrifuged at 5000 r/min (2817× *g*) for 15 min, and then, the free oil on the upper layer was sucked and weighed to calculate the free oil yield based on Equation (1). For the remaining mixture that had its free oil removed, the emulsion oil was removed, and the aqueous phase was carefully poured out; then, 5 mL of distilled water was added. The mixture was then washed twice and dried at 55 °C for 12 h to obtain peanut dregs. Peanut dregs were weighed to calculate the protein retention rate based on Equation (2) [6,20]. Peanut dregs were ground fine using a mortar, and then sealed and refrigerated at 4 °C for later use. Peanut dregs from unroasted peanuts were taken as the control. Similarly, peanut oil extraction was carried out using unroasted peanuts as raw materials and with water as an extractant to compare the effects of roasting and ethanol on the oil extraction efficiency. In addition, 50 of g peanut paste was used each run as raw material and extracted using aqueous ethanol to investigate the effect of roasting on the material distribution after extraction.

(1) $$\mathrm{Free}\;\mathrm{oil}\;\mathrm{yield}\;(\%)=\frac{\mathrm{free}\;\mathrm{oil}\;\mathrm{mass}\;(g)}{\mathrm{paste}\;\mathrm{mass}\;(g)\;\times \;\mathrm{oil}\;\mathrm{content}\;(\%)}\times \;100$$

(2) $$\mathrm{Protein}\;\mathrm{retention}\;\mathrm{rate}\;(\%)\;=\frac{\mathrm{dreg}\;\mathrm{mass}\;(g)\;\times \;\mathrm{dreg}\;\mathrm{protein}\;\mathrm{content}\;(\%)}{\mathrm{peanut}\;\mathrm{mass}\;(g)\;\times \mathrm{peanut}\;\mathrm{protein}\;\mathrm{content}\;(\%)}\times \;100$$

### 2.4. Protein Functional Properties

The soluble protein content was determined using the coomassie brilliant blue staining method [21]. The water(oil)-holding capacity was determined using the gravimetric method [22]. Emulsification activity and emulsion stability were determined using the turbidity method [23]. Foaming ability and foam stability were determined using the volumetric method [24]. Surface hydrophobicity was determined using the spectrophotometric method [25].

### 2.5. X-ray Diffraction (XRD)

The XRD pattern was analyzed using an XRD instrument (D8 series, Bruker Corporation, Karlsruhe, Germany). The sample was pressed on a stainless stub and placed in s sample chamber. The radiation was filtered through Cu-Kα nickel at a wavelength of 0.15 nm. The operated voltage and electric current were 40 kV and 40 mA, respectively. The scanning range, resolution ratio and scanning rate were respectively set to 4–70°, 0.02° and 8°/min [26] for the collection of the diffraction data.

### 2.6. Differential Scanning Calorimeter (DSC)

The thermal properties were determined using a DSC (DSC2500, TA Instruments, New Castle, DE, USA). A sample of 4.00 mg was accurately weighed into aluminum pans and evenly mixed with ultrapure water in a liquid-to-solid ratio of 4:1 (μL/mg). Then, the sample was hermetically sealed and placed at 4 °C for 24 h. Then, it was heated in the range from 30 °C to 120 °C at a scanning rate of 5 °C/min under a nitrogen atmosphere (50 mL/min) [27]. An empty aluminum pan was used as the control.

### 2.7. Ultraviolet (UV) and Intrinsic Fluorescence Spectra

The UV spectrum was analyzed using a UV-Vis spectrophotometer (ΜV-5800C, Metash Instruments, Shanghai, China). A barium sulfate and sample were mixed in a 1:2 (g/g) ratio, pressed on a stainless stub and placed in the sample chamber. A UV spectrum was obtained in the 200 nm–400 nm wavelength range with a 2.0 nm bandwidth and a 60 nm/min scan rate [28].

The intrinsic fluorescence spectrum was obtained using a fluorescence spectrophotometer (RF-5301PC, Shimadzu Corporation, Kyoto, Japan). The sample was dissolved in 0.1 mol/L of phosphate buffer at pH 7.0, followed by gradient dilution using distilled water. The intrinsic fluorescence spectrum was taken at an excitation wavelength of 280 nm (slit 3 nm), emission wavelength of 220 nm–600 nm (slit 3 nm) and scan rate of 10 nm/min [29].

### 2.8. Fourier-Transform Infrared Spectroscopy (FTIR)

A sample of 1 mg and potassium bromide of 100 mg dried at 130 °C for 4 h were mixed thoroughly in an agate mortar, grounded evenly and pressed on a tablet press machine at a pressure intensity of 10 T/cm^2^ to obtain a surface uniform, crack-free and translucent tablet. The FTIR spectroscopy was collected from 4000 cm^−1^ to 400 cm^−1^ over 512 scans (IRPrestige-21, Shimadzu Corporation, Kyoto, Japan), with a resolution of 2 cm^−1^ [30], and then baseline correction, smoothing and normalization were performed. The amide I band (1600–1700 cm^−1^) and amide III band (1220–1350 cm^−1^) spectra were used to analyze the secondary structure of the protein. The spectrum was dealt with PeakFit 4.12 software for region selection, Gaussian deconvolution and second-order derivative fitting to improve the spectral resolution. Finally, combined with the corresponding secondary structure identification, the relative content of each secondary structure was calculated using the ratio of peak areas [31].

### 2.9. Raman Spectroscopy

A laser confocal microscopy Raman spectrometer (HR-800, Horiba Jobin-Yvon Corporation, Paris, France) was switched to a 50× lens at room temperature (25 °C) and was used to collect the spectrum with argon ions at a laser wavelength of 514.5 nm. The laser power was set to 20 mW. Before measurement, the laser wavelength was calibrated at 520.7 nm using monocrystalline silicon. The diameter of the laser spot was about 1 μm focusing on the sample. The sample was placed on a glass slide and then focused using the laser; Raman spectra were recorded for at least three different points in the wavenumber range of 200–4000 cm^−1^. The spectra were collected under the following conditions: an exposure time of 60 s, resolution of 2 cm^−1^, sampling speed of 120 cm^−1^/min, with data recorded every 1 cm^−1^ [32].

### 2.10. Microscopy Observation

A small amount of sample (paste or powder) was picked up with a toothpick, evenly spread on a glass slide, stained with 0.5% oil red O for 3 min, covered with a cover glass, eluted with 25% ethanol twice and then observed under an optical microscope (LW200CA, Cewei Photoelectricity Technology Co., Ltd., Shanghai, China) at room temperature.

The sample was scattered evenly on a circular aluminum stub with double-sided sticky tape, sprayed gold to a thickness of 10 nm, and observed in a cold field emission scanning electron microscope (model S-4800, Hitachi Company, Tokyo, Japan) at an accelerating voltage of 5 kV.

### 2.11. Statistical Analysis

Each experiment was conducted in triplicate, and the results were expressed as the mean ± standard deviation. SPSS 19.0 (International Business Machines Corporation, Armonk, NY, USA) was used for one-way analysis of variance (ANOVA) and Duncan’s multiple range tests were used for identifying significant differences (*p* < 0.05), and Origin 2022 SR1 (OriginLab Corporation, Northampton, MA, USA) software was used for plotting.

## 3. Results and Discussion

### 3.1. Effects of Roasting and Ethanol on the Free Oil Yield

**Figure 1:**
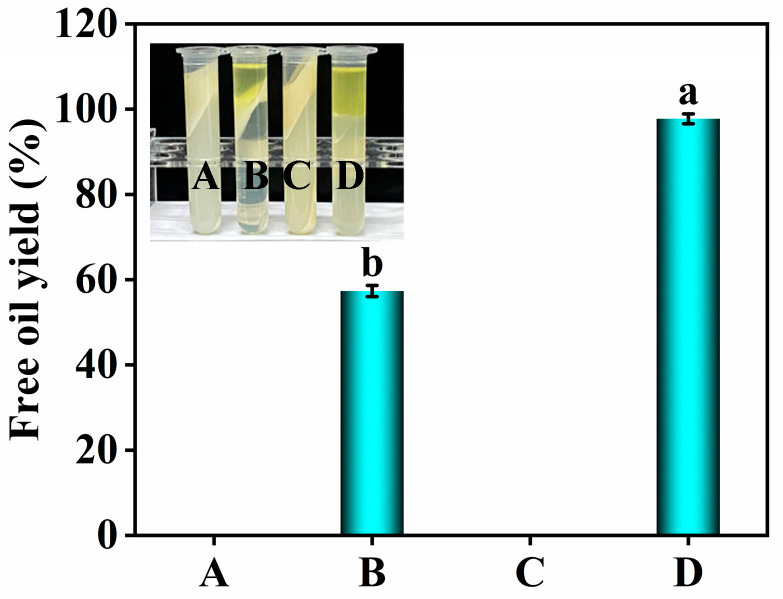
Effects of roasting and ethanol on the free oil yield. (A,B) represent using water and aqueous ethanol as extractants for unroasted peanuts, and (C,D) represent using water and aqueous ethanol as extractants for roasted peanuts. Different letters (a,b) indicate significant differences (*p* < 0.05).

The effects of roasting and ethanol on the free oil yield of peanuts are shown in Figure 1. Whether roasted or not, using only water as an extractant could not obtain free oil from peanuts (Columns A and C in Figure 1). In contrast, when aqueous ethanol was used as an extractant, a large amount of free oil could be obtained from the peanuts (Columns A and B in Figure 1), and roasting can significantly increase the free oil yield from 57.33% of the control to 97.74% (Columns B and D in Figure 1). Therefore, the roasting-assisted aqueous ethanol extraction of peanut oil was very efficient without the need for additional demulsification. In fact, it was the roasting intensity (the combination of temperature and time) that affected the extraction efficiency of peanut oil. Briefly, low temperatures required a longer time, while high temperatures required a shorter time. The ethanol played a key role in reducing the emulsification during the oil extraction, which may be due to its strong polarity in the aqueous phase. The significant reduction of interfacial tension made the oil-in-water emulsion in the system convert from stable to unstable [33], which led to the release of more free oil, increasing the free oil yield. More importantly, roasting and ethanol have a synergistic effect, which can efficiently release free oil from peanuts, and the extraction process was highly efficient. Meanwhile, the effects of roasting-assisted aqueous ethanol extraction on the structure and functional properties of peanut proteins were of great significance for the rational utilization of protein resources. Therefore, further research was needed to help reveal the underlying reasons for the increase in the free oil yield.

### 3.2. Effect of Roasting on the Material Distribution during Oil Extraction

The effect of roasting on the material distribution is shown in Table 1. After roasting, the proportion of free oil significantly increased, while the emulsified oil, aqueous phase oil and dreg residual oil all significantly decreased. The roasting treatment greatly promoted the dissolving out of the total oil and the release of free oil, and inhibited the formation of emulsified oil. The retained proteins in the dreg were significantly reduced, while the retained carbohydrates significantly increased, suggesting that roasting promoted the dissolution of proteins and reduced that of carbohydrates during the extraction process. This may be the reason for the significant decrease in dreg residual oil. In addition, the total solids in the aqueous phase were significantly reduced. Combined with the increase in dissolved protein, it was confirmed that roasting reduced the amount of carbohydrates dissolved. The above changes can explain the increase in the free oil yield from the perspective of material distribution, i.e., the increase in dissolved protein and the decrease in dissolved carbohydrates resulted in a decrease in the emulsification degree and dreg residual oil. Moreover, the protein retention rate of the dreg after oil extraction reached 75.80%, which greatly avoided the recycling difficulty and waste of peanut protein resources.

### 3.3. Effect of Roasting on the Functional Properties of Dreg Proteins

In general, severe denaturation can lead to a decrease or even loss of protein functional properties. On the other hand, moderate denaturation is beneficial for reducing the protein surface hydrophobicity [34], thus increasing hydrophilicity. The effect of roasting on the functional properties of dreg proteins is shown in Table 2. Before and after roasting, there was no significant difference in the soluble protein content for the obtained peanut dreg. The lower values of soluble protein were due to the fact that most soluble proteins were dissolved when aqueous ethanol was used as the extraction agent [29]. However, due to the reduction in the dreg mass after roasting treatment (Footnote in Table 1), the retained amount of soluble protein in the roasted peanut dreg decreased, suggesting that roasting promoted the dissolution of proteins during the extraction process, which may be attributed to a slight denaturation of proteins. This is beneficial for the oil release from the oilseed tissue into the aqueous phase.

After roasting, the water-holding capacity of dreg proteins was significantly improved (135.33%), while the oil-holding capacity was significantly reduced (95.26%), which reduced the binding ability with oil and was beneficial for diminishing the dreg residual oil. Roasting also significantly decreased the emulsion activity and stability, which helped to hinder the formation and stability of emulsified oil during the extraction process, thereby increasing the free oil yield. In addition, the foaming ability and stability significantly decreased after roasting, which may be a result of more hydrophobic groups buried inside the molecule [35]. The less binding amount between the protein and bromophenol blue, the smaller the surface hydrophobicity of the protein [25]. After roasting, the bromophenol blue binding amount of peanut dreg proteins significantly decreased, showing a lower surface hydrophobicity and higher hydrophilicity; this can also explain the significant decrease in the dreg residual oil.

Overall, the functional properties of dreg proteins changed to a certain extent after roasting, which was not only helpful to the dissolving out of the oil from the oilseed tissue into the aqueous phase, but also unfavorable to emulsion formation, thus releasing more free oil. On the other hand, the functional properties of peanut dreg proteins were retained to a large extent, especially in that the soluble protein content exhibited no significant difference from that before roasting. The retention rate was 91.82% for the foaming ability, 79.73% for the emulsion activity, 76.48% for the foaming stability and 62.57% for the emulsion stability. Therefore, the peanut dreg proteins from roasting-assisted aqueous ethanol extraction still have good utilization values.

### 3.4. Effect of Roasting on the XRD Pattern

**Figure 2:**
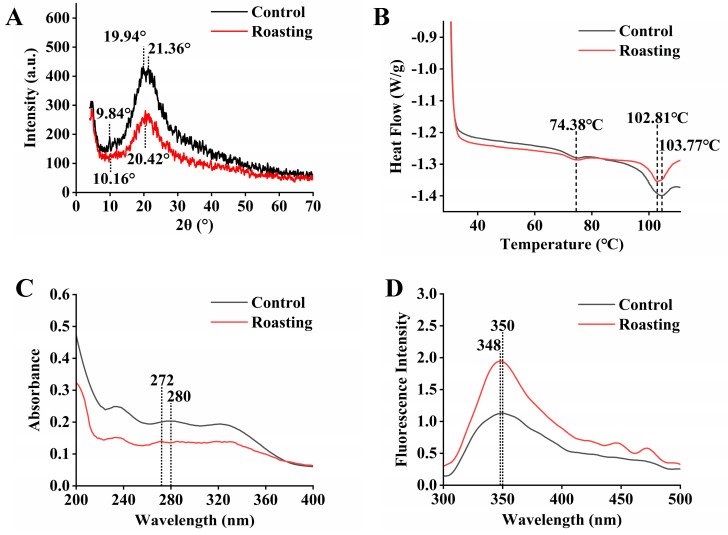

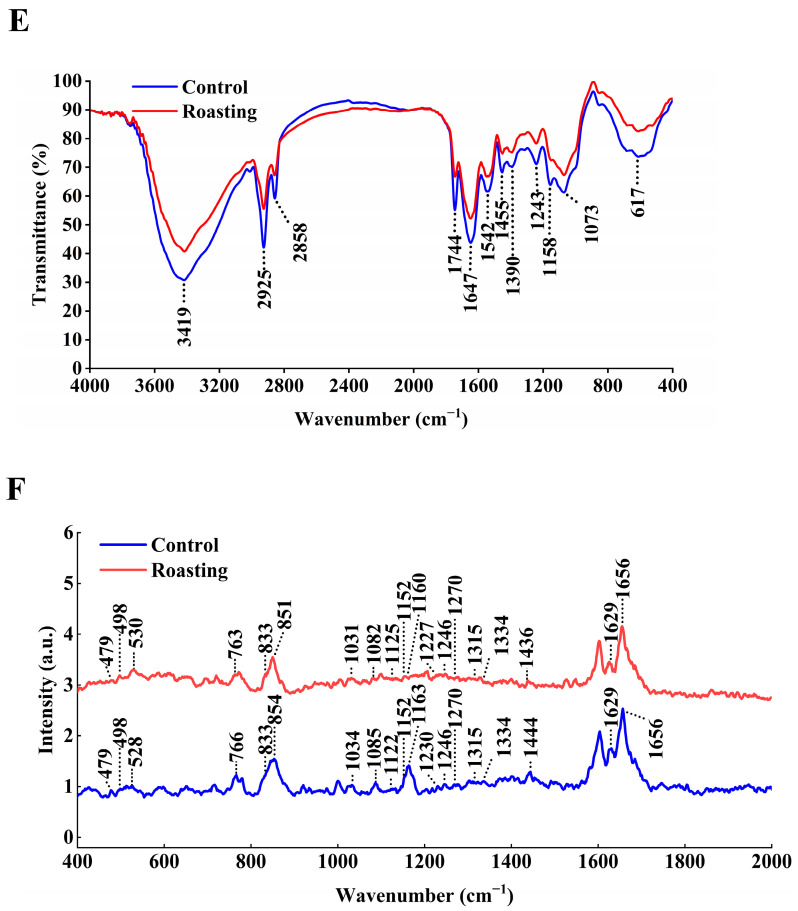
Structural characterization of peanut dreg proteins. (**A**–**F**) represent the XRD pattern, DSC diagram, UV spectrum, intrinsic fluorescence spectrum, FTIR spectroscopy and Raman spectroscopy, respectively.

XRD is commonly used to study changes in the crystal structure of protein molecules [26]. The XRD spectrum of peanut dreg proteins is shown in Figure 2A. Before roasting, diffraction peaks of the dreg proteins appeared at 9.84° and 19.94°/21.36°, corresponding to the secondary structure of the α-helix and β-sheet structures [36], respectively. After roasting, the diffraction intensity of α-helix significantly decreased, while the double diffraction peaks of β-sheet transformed into a single diffraction peak (20.42°), suggesting that roasting made the molecular structure of peanut dreg proteins more stretched [37]. In addition, the relative crystallinity was obtained by analyzing the areas of the crystallization zone and total diffraction spectrum. After roasting, the relative crystallinity of the dreg proteins decreased from 56.15% to 49.91%, with a retention rate of 88.89% compared to the control. This indicated that roasting caused the denaturation of the dreg proteins to a certain extent, but the degree of denaturation was relatively low. But it cannot be determined which amino acid has undergone oxidative denaturation. Subsequent FTIR and Raman spectra may be used for further analysis.

### 3.5. Effect of Roasting on the Thermal Properties

The DSC can reflect the structural and conformational changes in proteins by the denaturation temperature (T_d_) and enthalpy (ΔH), conveniently measuring the thermal stability of proteins [38]. The peak position represents the denaturation temperature of proteins, and the enthalpy value represents the bond energy that maintains the folding conformation of proteins. For the control, the heat flow diagram of peanut dreg proteins displays two denaturation temperatures at 74.38 °C and 103.77 °C (Figure 2B), respectively, representing peanut conarachin for the former and peanut arachin for the latter. After the roasting treatment, there was no significant change in the denaturation temperature or enthalpy of conarachin. Although the denaturation temperature of arachin did not change significantly, the enthalpy value decreased from 5.52 J/g to 4.40 J/g (79.71%), suggesting a low degree of denaturation induced by the roasting treatment.

### 3.6. Effect of Roasting on Ultraviolet and Intrinsic Fluorescence Spectra

The hydrophobic amino acids of proteins, such as tryptophan, tyrosine and phenylalanine, have side chains containing indole rings, phenolic hydroxyl groups or phenyl groups, and the maximum absorption peaks in ultraviolet spectra commonly appear at 260 nm, 275 nm or 280 nm [28], respectively. The microenvironment changes in hydrophobic amino acid residues can alter the molecular conformation, resulting in peak shifts and intensity changes. The absorption peak of dreg proteins at 280 nm shifted to 272 nm after roasting (Figure 2C) but had no significant change in peak intensity. This indicates that the peptide chains of the protein molecules became more stretched [39], and the hydrophobicity of the microenvironment near tryptophan or tyrosine residues was reduced [39].

Due to the presence of benzene rings or conjugated double bonds in tryptophan, tyrosine or phenylalanine residue, proteins can generate endogenous fluorescence at specific excitation wavelengths, with fluorescence emission peaks occurring near 348 nm, 303 nm or 282 nm [40]. In addition, tyrosine may be ionized, and its fluorescence can almost be completely quenched when approaching carboxyl or amino groups [41]. The quantum yield of phenylalanine is relatively low, and it produces almost no fluorescence [41]. Therefore, an excitation wavelength of 280 nm was chosen to obtain a fluorescence spectrum, at which tryptophan would be excited. The fluorescence peak near 348 nm represents the changes in the microenvironment of tryptophan, indirectly suggesting the changes in the protein molecular conformation [42]. After roasting, the fluorescence peak position and width of tryptophan residues remained unchanged (Figure 2D), but the peak shape became sharper, and the fluorescence intensity was significantly enhanced. This indicates that the spatial conformation of dreg proteins changed [43], resulting in a more compact molecular structure. Subsequent scanning electron microscopy morphologies may be used for further analysis. At the same time, tryptophan residues were buried inside the molecule [43], and the surface hydrophobicity was reduced. This was consistent with the measuring results of the surface hydrophobicity (Table 2), which was helpful to reduce the binding between proteins and oils and thus promote the release of free oil.

### 3.7. Effect of Roasting on FTIR Spectroscopy

FTIR spectroscopy is used to characterize changes in the secondary structures of proteins, and it can also be used to analyze the changes in certain functional groups and microenvironments [44]. The FTIR spectroscopy and characteristic frequency assignments of peanut dreg proteins are shown in Figure 2E and Table S1, respectively. The absorption peaks in the ranges of 1600–1700 cm^−1^, near 1542 cm^−1^ and 1220–1350 cm^−1^ represent the amide I, II and III bands of proteins, respectively. They are commonly used to reflect changes in protein chemical bond vibrations, where the amide I band typically represents the stretching vibration of C=O and C-N [45], and the amide III band typically represents the stretching vibration of C-N and the bending vibration of N-H [46]. After roasting, the absorption peak positions and intensities of the amide I, II and III bands showed no significant changes, suggesting that the protein conformation did not undergo significant alteration. In addition, the absorption peaks at 1073 cm^−1^ and 1158 cm^−1^ represent the characteristic absorption of sulfinic acid and sulfoxide [47]. After roasting, no significant change appeared for the former, while the latter showed a blue shift toward 1153 cm^−1^ in peak position and a significant decrease in intensity, suggesting a decrease in the sulfoxide of proteins, which may be due to the decomposition of the oxidation products of sulfur-containing amino acid residues [47]. The absorption peaks at 1390 cm^−1^ and 1744 cm^−1^ both represent C=O stretching vibrations. After roasting, the former had no significant change, while the latter showed a significant decrease in intensity, suggesting that carbonyl groups may become more bound. The absorption peak at 1455 cm^−1^ represents aliphatic hydrocarbons and benzene rings, no significant changes appeared after roasting. The absorption peaks at 2858 cm^−1^ and 2925 cm^−1^ represent the C-H stretching vibrations of the methyl and methylene of proteins. After roasting, the peak positions of both remained unchanged, but the intensity of the latter significantly decreased, showing a decrease in the freedom degree of methylene. The peak intensity near 3419 cm^−1^, which represents the O-H and N-H stretching vibrations and hydrogen bonding [48], significantly decreased, and the width narrowed, suggesting a reduction in the degree of hydrogen bonding after roasting.

The spectra of the amide I and III bands were selected to analyze protein secondary structure changes. For the amide I band, the peaks at 1621 cm^−1^, 1622 cm^−1^ and 1637 cm^−1^ represent β-sheet, the peak at 1655 cm^−1^ represents α-helix and the peaks at 1670 cm^−1^, 1672 cm^−1^, 1685 cm^−1^ and 1687 cm^−1^ represent β-turn [46]. For the amide III band, a peak range of 1220–1250 cm^−1^ represents β-sheet, that of 1245–1270 cm^−1^ represents random coil, that of 1265–1295 cm^−1^ represents β-turn and that of 1290–1330 cm^−1^ represents α-helix [49].

The peak fitting results of the amide I and III bands of dreg proteins are shown in Figure S1. Before roasting, the amide I band of dreg proteins could be divided into five peaks (Figure S1A). After roasting, the numbers and positions of the fitting peaks from the amide I band remained unchanged (Figure S1B). A similar situation occurred for the amide III band of dreg proteins. Before roasting, the amide III band of dreg proteins could be divided into seven peaks (Figure S1C), and the numbers and positions of the fitting peaks remained unchanged after roasting (Figure S1D). Thereafter, the fitted peak areas of the amide I and III bands were calculated to obtain the relative content of individual secondary structures, as shown in Table 3.

After roasting, the α-helix structure significantly decreased, and the β-fold, β-turn and random coil structures significantly increased. Whether roasted or not, β-sheet was the most abundant secondary structure of dreg proteins. The decrease in the α-helix structure was possibly due to a destruction of hydrogen bonds after roasting, or due to the polymerization of protein side-chain groups, which all destroyed the stability of α-helix [31]. From the perspective of the changes in the secondary structure contents, dreg proteins underwent a certain degree of denaturation, but the degree of denaturation was relatively low.

### 3.8. Effect of Roasting on Raman Spectroscopy

Raman spectroscopy can be used to study non-polar groups of proteins, reflecting changes in the microenvironment of amino acid side chains, thereby providing information on protein structures [32]. The Raman spectra of dreg proteins are shown in Figure 2F. The peak near 1629 cm^−1^ represents the β-sheet within the amide I band, and the peaks near 1230 cm^−1^ and 1246 cm^−1^ represent the β-sheet within the amide III band. After roasting, the intensities of the above peaks did not change significantly. The Raman intensity of the C-H bond at 1444 cm^−1^ significantly decreased after roasting, suggesting a reduction in the C-H bending vibration [32]. After roasting, the Raman intensities of the disulfide bonds and sulfoxides at 1085 cm^−1^ significantly decreased, suggesting that the oxidation products of sulfur-containing amino acid residues in the proteins were further decomposed to a certain extent [50]. However, the peak of sulfone at 1163 cm^−1^ almost disappeared, suggesting that the sulfone could be further oxidized. It was inferred that methionine and cystine had undergone oxidative denaturation to a certain extent. The Fermi doublet at 854 cm^−1^ and 833 cm^−1^ represents tyrosine resonances of proteins, and their intensity ratio significantly decreased after roasting, suggesting that the tyrosine residues were more buried [51]. The Raman peak near 766 cm^−1^ represents the tryptophan indole ring of proteins, and the peak intensity significantly decreased after roasting, suggesting that tryptophan residues were also more buried [50]. The above results indicate that sulfur-containing and aromatic amino acids in the dreg proteins underwent a certain degree of oxidation after roasting.

### 3.9. Effect of Roasting on the Microemulsion Morphology of Peanut Paste

**Figure 3:**
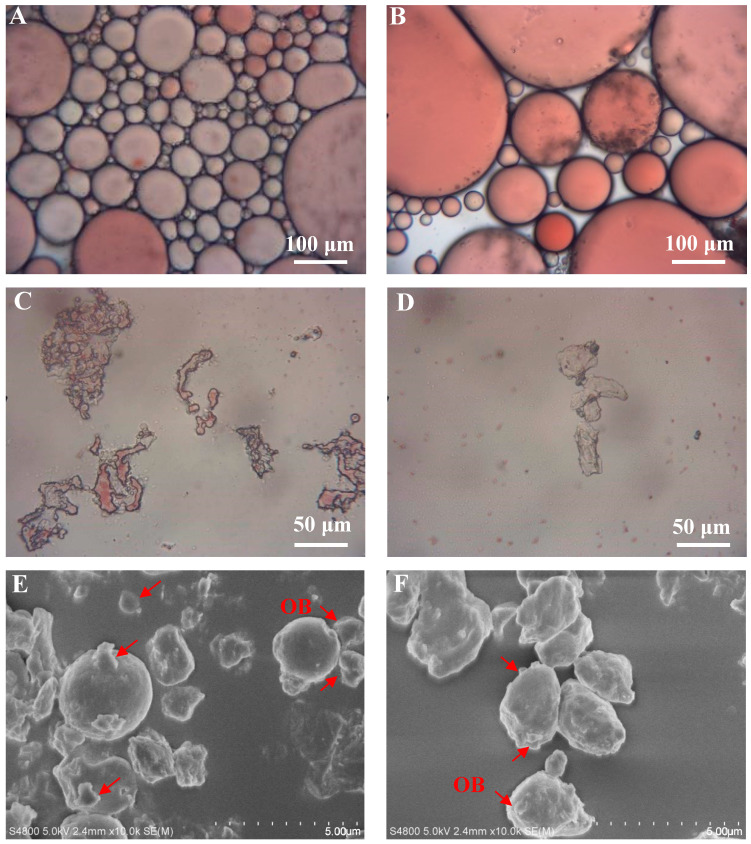
Morphologies of peanut paste microemulsion and peanut dregs. (**A**,**B**) represent the microemulsion morphologies of peanut paste before and after roasting, (**C**,**D**) represent the light microscopy morphologies of peanut dregs stained with oil red O before and after roasting, and (**E**,**F**) represent the scanning electron microscopy morphologies of peanut dregs before and after roasting. OB represents oil body (where the red arrow points).

Before roasting, the oil droplets were smaller, the degree of oil body fusion was lower, and the red color looked dim (Figure 3A), suggesting that the oil bodies in the unroasted peanut tissue exist in the form of microemulsion. After roasting, the size of the oil droplets significantly increased, the degree of oil body fusion improved, and the red color looked bright (Figure 3B), suggesting that the microemulsion state was broken to a large extent, which was helpful for the separation of oil from the proteins and the formation of free oil.

### 3.10. Effect of Roasting on the Micromorphology of Peanut Dregs

The peanut dreg particles were larger before roasting treatment, a few cell structures were not completely disintegrated, and most of the particles showed obvious residual oil, which had been stained red by oil red O (Figure 3C). The peanut dregs obtained after roasting had smaller particle sizes, with only a small amount of large particles remaining, but there was almost no obvious residue oil with red spots (Figure 3D).

In addition, from the morphology of scanning electron microscopy, there were still some larger oil bodies adsorbed on the surface of the protein bodies in the unroasted control, and they were partially wrapped. The shapes of the protein bodies were relatively regular before roasting, showing a smooth spherical surface (Figure 3E). After roasting, the visible oil bodies on the surface of protein bodies were very small and few (Figure 3F), which may be attributed to the increased water-holding capacity and surface hydrophilicity of the proteins, making it easier to separate from oil bodies. Moreover, the shapes of protein bodies became irregular and ellipsoidal, with a collapsed surface.

## 4. Conclusions

This study aimed to investigate the effect of the roasting-assisted aqueous ethanol extraction of peanut oil on the structure and functional properties of dreg proteins, to reveal the mechanism of improving the extraction efficiency of peanut free oil and to provide a basis for the rational utilization of peanut protein resources. The results show that the roasted-assisted aqueous ethanol extraction could efficiently obtain free oil and dreg proteins from peanuts, accompanied by a high retention rate in the protein’s functional properties. The reason for the improvement of the free oil yield was that roasting promoted the fusion of oil bodies in peanut tissues; meanwhile, a low degree of the denaturation of peanut proteins resulted in an increase in the dissolving out of proteins to the aqueous phase. Moreover, the water-holding capacity of proteins increased, while the oil-holding capacity and surface hydrophobicity decreased, promoting the dissolving out and release of free oil. Using aqueous ethanol as an extractant, combined with roasting, helped to reduce the emulsification. The free oil yield of peanuts in this process could reach 97.74%, without the need for additional demulsification. These results are beneficial to the full utilization of oil and protein resources in peanut kernels. The whole process is green, environmentally friendly and safe, reducing the potential risks of air pollution, explosion, fire and neurotoxicity compared to traditional oil extraction methods, with broad application prospects.

## Figures and Tables

**Table 1:** Effect of roasting on the material distribution during oil extraction.

Treatment	FO (g)	EO (g)	APO (g)	APS (g)	DRO (g)	DP (g)	DC (g)	DM (g)
Control	27.99 ± 0.07 ^b^	16.32 ± 0.11 ^a^	1.36 ± 0.17 ^a^	9.40 ± 0.71 ^a^	3.74 ± 0.21 ^a^	19.37 ± 0.22 ^a^	20.19 ± 0.47 ^b^	1.64 ± 0.02 ^a^
Roasting	47.34 ± 0.16 ^a^	0.33 ± 0.19 ^b^	0.63 ± 0.11 ^b^	8.27 ± 0.12 ^b^	1.10 ± 0.11 ^b^	17.78 ± 0.05 ^b^	22.87 ± 0.10 ^a^	1.68 ± 0.01 ^a^

Note: FO, EO, APO, APS, DRO, DP, DC and DM represent free oil, emulsified oil, aqueous phase oil, aqueous phase solid, dreg residual oil, dreg protein, dreg carbohydrate and dreg moisture, respectively. The dreg quality of the control was 44.94 ± 0.51 ^a^ g, and that of roasting treatment was 43.43 ± 0.11 ^b^ g. Different letters (a,b) in the same column indicate significant differences (*p* < 0.05).

**Table 2:** Effect of roasting on the functional properties of dreg proteins.

Treatment	SP (%)	WHC (g/g)	OHC (g/g)	EA (m^2^/g)	ES (m^2^/g)	FA (%)	FS (%)	S_0_ (μg)
Control	19.28 ± 0.02 ^a^	5.69 ± 0.12 ^b^	5.06 ± 0.03 ^a^	8.98 ± 0.32 ^a^	68.34 ± 0.15 ^a^	16.25 ± 0.42 ^a^	55.18 ± 0.13 ^a^	72.15 ± 4.94 ^a^
Roasting	19.18 ± 0.15 ^a^	7.70 ± 0.03 ^a^	4.82 ± 0.10 ^b^	7.16 ± 0.09 ^b^	42.76 ± 0.52 ^b^	14.92 ± 0.63 ^b^	42.20 ± 0.37 ^b^	38.57 ± 1.46 ^b^

Note: SP, WHC, OHC, EA, ES, FA, FS and S_0_ represent soluble protein, water-holding capacity, oil-holding capacity, emulsion activity, emulsion stability, foaming ability, foaming stability and surface hydrophobicity, respectively. Different letters (a,b) in the same column indicate significant differences (*p* < 0.05).

**Table 3:** Relative content of secondary structures from amide I and III bands of dreg proteins.

Treatment	α-Helix (%)	β-Sheet (%)	β-Turn (%)	Random Coil (%)
Control	17.52 ± 3.52 ^a^	30.12 ± 2.09 ^b^	28.24 ± 4.08 ^b^	24.12 ± 1.99 ^b^
Roasting	1.29 ± 0.25 ^b^	34.79 ± 3.12 ^a^	31.19 ± 0.85 ^a^	32.73 ± 2.31 ^a^

Note: Different letters (a,b) in the same column indicate significant differences (*p* < 0.05).

## Data Availability

The original contributions presented in the study are included in the article/supplementary material, further inquiries can be directed to the corresponding author.

## Supplementary Materials

The following supporting information can be downloaded at https://www.mdpi.com/article/10.3390/foods13050758/s1.
