# Supplementary material for: Effects of the Roasting-Assisted Aqueous Ethanol Extraction of Peanut Oil on the Structure and Functional Properties of Dreg Proteins
Source: Foods. 2024 Feb 29;13(5):758. doi: 10.3390/foods13050758 (PMC10930452; doi:10.3390/foods13050758)
Supplement: Supplementary file 1 [file foods-13-00758-s001.zip › Figure S1.pdf]

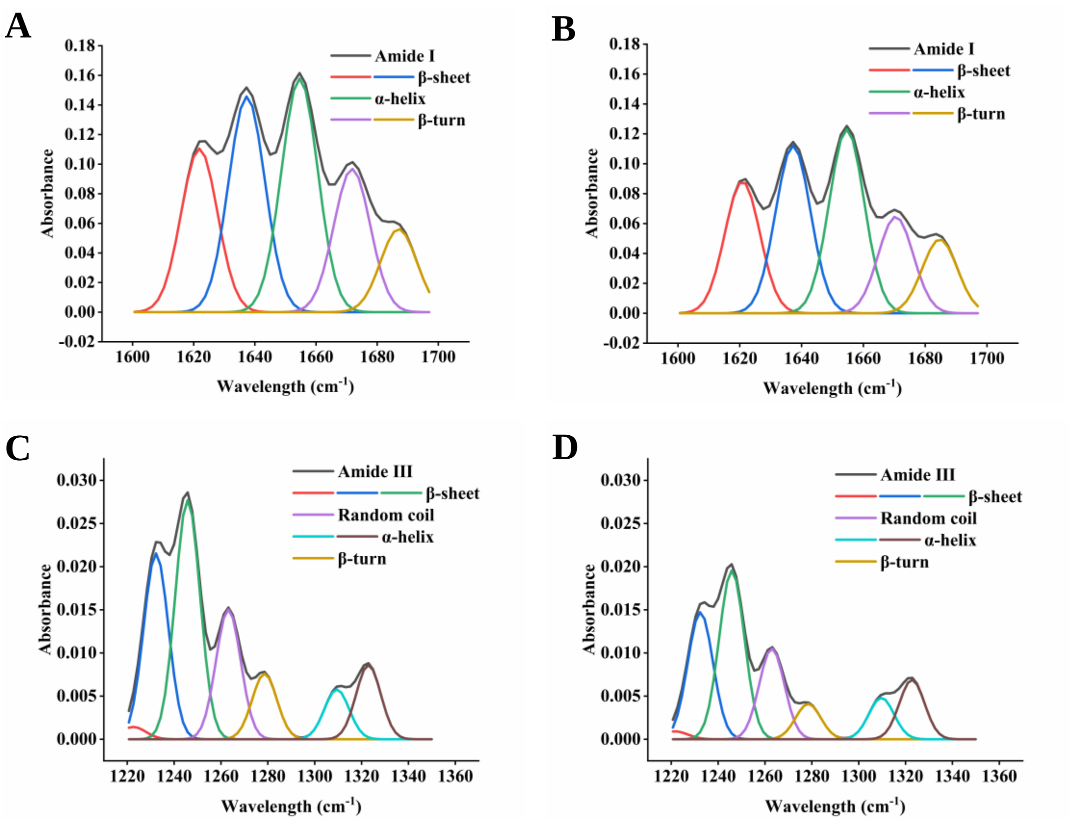

Figure S1. Fitted spectra of the amide I and III bands of dreg proteins. The letters of (A–B) represent the amide I bands before and after roasting, respectively. The letters of (C–D) represent the amide III bands before and after roasting, respectively.
