# Supplementary material for: Effects of the Roasting-Assisted Aqueous Ethanol Extraction of Peanut Oil on the Structure and Functional Properties of Dreg Proteins
Source: Foods. 2024 Feb 29;13(5):758. doi: 10.3390/foods13050758 (PMC10930452; doi:10.3390/foods13050758)
Supplement: Supplementary file 1 [file foods-13-00758-s001.zip › Table S1.pdf]

Table S1. FTIR characteristic frequency and tentative assignment of dreg proteins.

Band position (cm^{-1})		Assignment	Normalized intensity (A/A_{1654})	
Control	Roasting		Control	Roasting
1073	1072	O-S-OH	0.60A	0.61A
1158	1153	S=O, C-O	0.55A	0.50B
1243	1242	Amide III	0.42A	0.38A
1390	1392	C=O	0.43A	0.44A
1455	1454	Aliphatic hydrocarbon, benzene ring	0.47A	0.45A
1542	1542	Amide II	0.59A	0.62A
1647	1649	Amide I	1.01A	1.01A
1744	1746	C=O	0.72A	0.63B
2858	2859	CH_3	0.64A	0.62A
2925	2926	CH_2	1.06A	0.91B
3419	3417	O-H, N-H, Hydrogen bond	1.44A	1.39B

Note: Different letters (A-B) in the same row indicate significant differences ($p < 0.05$).
